# Assessing the methodological strengths and limitations of the Spanish Society of Medical Oncology (SEOM) guidelines: a critical appraisal using AGREE II and AGREE-REX tool

**DOI:** 10.1007/s12094-023-03219-0

**Published:** 2023-06-27

**Authors:** Marilina Santero, Júlia de Mas, Berta Rifà, Inés Clavero, Irene Rexach, Xavier Bonfill Cosp

**Affiliations:** 1 Universitat Autònoma Barcelona (UAB), Barcelona, Spain; 2 Iberoamerican Cochrane Centre, Biomedical Research Institute Sant Pau (IIB Sant Pau), Barcelona, Spain; 3 CIBER Epidemiología y Salud Pública (CIBERESP), Barcelona, Spain

**Keywords:** Practice guidelines as topic, Cancer, Review, Medical oncology, Evidence-based medicine, Quality assessment

## Abstract

**Background:**

The Spanish Society of Medical Oncology (SEOM) has provided open-access guidelines for cancer since 2014. However, no independent assessment of their quality has been conducted to date. This study aimed to critically evaluate the quality of SEOM guidelines on cancer treatment.

**Methods:**

Appraisal of Guidelines for Research and Evaluation II (AGREE II) and AGREE-REX tool was used to evaluate the qualities of the guidelines.

**Results:**

We assessed 33 guidelines, with 84.8% rated as “high quality”. The highest median standardized scores (96.3) were observed in the domain “clarity of presentation”, whereas “applicability” was distinctively low (31.4), with only one guideline scoring above 60%. SEOM guidelines did not include the views and preferences of the target population, nor did specify updating methods.

**Conclusions:**

Although developed with acceptable methodological rigor, SEOM guidelines could be improved in the future, particularly in terms of clinical applicability and patient perspectives.

**Supplementary Information:**

The online version contains supplementary material available at 10.1007/s12094-023-03219-0.

## Introduction

Published cancer research is increasing rapidly [1], and having trustworthy health recommendations accessible is in high demand by guideline users [2]. For decision-makers, clinical practice guidelines (CPGs) have emerged to be a reference to improve the quality of care by bringing together the best available scientific evidence into specific recommendations to improve the quality of care [3]. Their development involves identifying and refining a subject area, forming a multidisciplinary panel of experts, conducting a systematic review of the evidence, formulating recommendations based on the evidence, and grading the strength of the recommendations [4]. All the process should be transparent, evidence-based, and involve stakeholder input [5].

The European Society for Medical Oncology (ESMO) [6], the American Society of Clinical Oncology (ASCO) [7] and the Spanish Society of Medical Oncology (SEOM) [8] guidelines are prepared and reviewed by leading experts and they provide a set of evidence-based recommendations to serve as a guide for healthcare professionals and outline appropriate methods of treatment and care. Particularly, SEOM is a national, non-profit scientific society that promotes studies, training, and research activities. SEOM wants to increase its role as a reference society, a source of opinion, and rigorous knowledge about cancer for all the agents involved, patients, and society in general [8]. The SEOM’s project to develop guidelines started in 2010 as a perceived need by the Spanish oncologists, that required clinical practice documents tailored to the peculiarities of the Spanish healthcare system. Since 2014, open-access CPGs are available to facilitate clinical practice providing an eminently practical view of the most relevant considerations concerning various cancer-related scenarios [9].

To date, no independent assessment of its quality has been made, despite reports indicating that critical reviews of guidelines worldwide show they are not sufficiently robust [10, 11]. The quality of CPGs can be assessed using various tools, such as AGREE II and AGREE-REX, which evaluate the methodological quality, rigor, and transparency of guideline development. These tools can help to identify areas for improvement in CPGs and ensure that they are evidence-based and relevant to clinical practice. In this context, this study aims to critically assess the quality of CPGs on cancer treatment published by SEOM since 2014.

## Methods

### Study design

This is a critical review of CPGs. We followed rigorous standards [12] and reported our results according to the Preferred Reporting Items for Systematic Review and Meta-Analysis (PRISMA) 2020 checklist [13] (Supplementary file 1). Before starting the review process, we published a research protocol online in the Open Science Framework (OSF) repository [14].

### Eligibility criteria

We considered a definition for CPGs previously reported by the Institute of Medicine (IOM) [15]. The inclusion criteria (all criteria required) were as follows: (a) CPGs for cancer treatment; (b) supported by SEOM; (c) published in English, or Spanish; and (d) published from 2014 onwards.

Exclusion criteria (any criterion required) were: (a) CPGs on cancer prevention, screening, detection, diagnostic, mapping, staging, imaging, scanning, or follow-up without treatment recommendations; (b) CPGs not containing recommendations for specific cancer (pathology-related guidelines); or (c) unavailable papers, surveys, audits, editorials, letters to the editor, case reports or notes.

### Literature search

In February 2022, we identified eligible CPGs through electronic searches on MEDLINE (via PubMed), the SEOM website, and the Clinical and Translational Oncology Journal website, an international journal where SEOM guidelines are published. The search strategy for MEDLINE is presented in Supplementary file 2.

### Screening and data extraction

Two reviewers performed an independent title and abstract screening and full text afterwards. A third reviewer solved disagreements. We used Rayyan^®^, a free web-based software tool for conducting reviews [16]. Two reviewers extracted data independently from included guidelines, in a previously piloted form. We extracted a minimum dataset considering the general characteristics of included CPGs. Also, we extracted data for the development of a recommendation mapping. The lack of agreements was resolved through discussion until a consensus was reached. When we found more than one guideline for a specific cancer (also of different guideline versions), we decided to create a publication thread analyzing the CPGs as a whole with all its references.

### Quality assessment

Three independent reviewers assessed the quality of included CPGs using the AGREE II tool [17], developed by the International Appraisal of Guidelines, Research and Evaluation (AGREE) research team. The tool has become a widely used standard for evaluating the methodological quality and transparency of CPGs internationally [18]. The reviewers rated 23 key items across six domains: (1) scope and purpose; (2) stakeholder involvement; (3) rigor of development; (4) clarity of presentation; (5) applicability; and (6) editorial independence. Each item, including the two global rating items, was rated on a 7-point scale (1—strongly disagree to 7—strongly agree). As a complement to AGREE II, only for the guidelines scored as “high quality”, we used the instrument AGREE-REX, a new tool designed in 2019 to evaluate and optimize the clinical credibility and implementability of CPGs recommendations [19, 20]. AGREE-REX includes three key quality domains: clinical applicability (domain 1), values and preferences (domain 2), and implementability (domain 3), comprising 9 items that must be considered to ensure that guidelines recommendations are of high quality. This tool was used by the same three independent authors on a 7-point scale (1—strongly disagree to 7—strongly agree). Furthermore, the evaluator was asked about the recommendation of this guideline in the appropriate context or in the reviewers’ context. All the assessments were performed independently and blinded using an internally piloted data extraction spreadsheet in Microsoft Excel 2019.

### Statistical analysis

As suggested by the AGREE II instructions, we calculated domain scores as a sum of the average scores of individual items from all evaluators’ assessments in the domains. Then, we expressed the total scores for each domain as a percentage of the maximum possible score for that domain. Therefore, the range of possible scores was 0–100%, representing the worst and best possible ratings for each domain, respectively. Each domain assessed with a score of ≥ 60% was considered effectively addressed. We considered CPGs as “high quality” if they scored ≥ 60% in at least three of six AGREE II domains, including domain 3. If three domains or more were assessed with a score of  ≥ 60%, except domain 3, they were considered to be of “moderate quality” overall quality. Finally, CPGs scored < 60% in two or more domains and scored < 50% in domain 3 were considered “low quality”.

We performed all statistical analyses in RStudio [21], including boxplot and ggplot2 packages. Descriptive analyses were performed by estimators’ central tendency and dispersion including mean and standard deviation (SD) or median and interquartile ranges (IQR). We calculated inter-rater reliability using the average intraclass correlation coefficient (ICC) (two-way random mixed model), including the 95% confidence interval. ICC can be interpreted as follows, 0–0.2 (poor agreement); 0.3–0.5 (fair agreement); 0.5–0.75 (moderate agreement); 0.75–0.9 (strong agreement); and > 0.9 (almost perfect agreement).

## Results

**Fig. 1:**
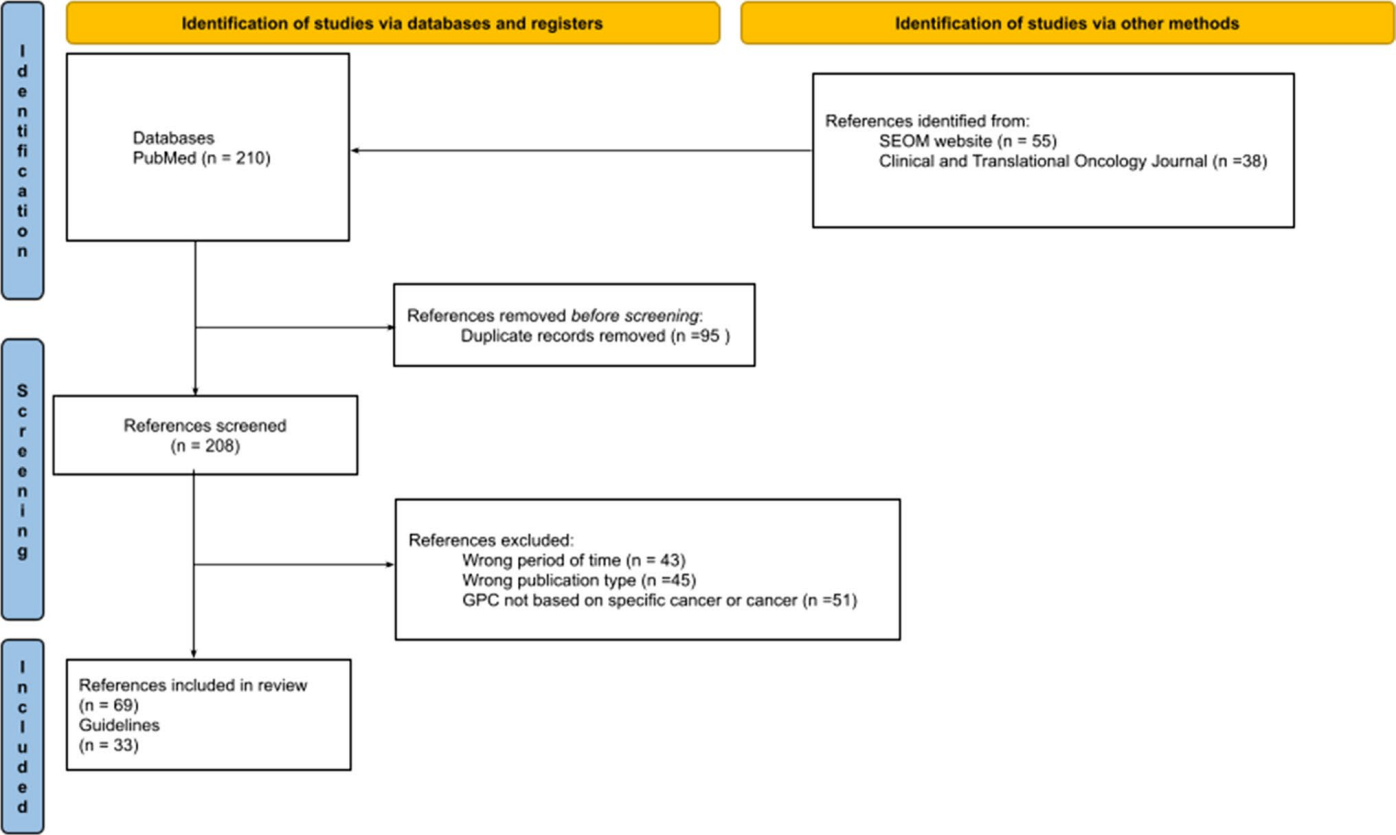
PRISMA 2020 flowchart. From: Page MJ, McKenzie JE, Bossuyt PM, Boutron I, Hoffmann TC, Mulrow CD, et al. The PRISMA 2020 statement: an updated guideline for reporting systematic reviews. BMJ 2021;372:n71. 10.1136/bmj.n71. For more information, visit: http://www.prisma-statement.org/

The PRISMA statement flow diagram (Fig. 1) depicts the flow of information through the different phases of our critical review. Finally, 208 relevant references remained for the full-text review, and 69 met the inclusion criteria for detailed analysis (excluded studies are presented in Supplementary file 3). After considering the subsequent updates of the same CPGs, a total of 33 CPGs were included [22–54].

Table 1 presents the characteristics of the reviewed CPGs. We identified 25 cancer types. Colorectal and breast cancer were the locations with more publications. On average, SEOM published seven guidelines per year. The year that witnessed the highest production was 2015 (13 guidelines), while 2017 had the lowest production (no guidelines published). Among the identified guidelines, 28 had been published over three years ago.

**Table 1:** Characteristics of included clinical practice guidelines by the Spanish Society of Medical Oncology (SEOM) (*n* = 33)

Cancer type	Guidelines [references]	Collaborative developers
Breast	SEOM clinical guidelines in advanced and recurrent breast cancer (2018)* [27, 60]	GEICAM, SOLTI
	SEOM clinical guidelines in early stage breast cancer (2018) [23, 55]*	GEICAM, SOLTI
	SEOM clinical guidelines in hereditary breast and ovarian cancer (2019)* [38, 70]	Sección SEOM de Cáncer Hereditario
Cervical	SEOM clinical guidelines for cervical cancer (2019)* [29, 82]	GEICO
Colorectal	SEOM clinical guidelines for diagnosis and treatment of metastatic colorectal cancer (2018)* [33, 66]	GEMCAD, TTD
	SEOM clinical guideline on hereditary colorectal cancer (2019)* [39, 65]	Sección SEOM de Cáncer Hereditario
	SEOM-GEMCAD-TTD clinical guidelines for localized rectal cancer (2021)* [26, 59]	GEMCAD, TTD
Endometrial	SEOM GEICO clinical guidelines on endometrial cancer (2021)*** [25, 57, 58]	GEICO
Gastrointestinal, Gastric or gastroesophageal junction	SEOM Clinical Guideline for gastrointestinal sarcomas (GIST) (2016) [48]	GEIS
	SEOM clinical guideline for the diagnosis and treatment of gastric cancer (GC) and gastroesophageal junction adenocarcinoma (GEJA) (2019)*** [44,67–69]	GEIS, GEMCAD, TTD
Glioblastoma	SEOM clinical guidelines for diagnosis and treatment of glioblastoma (2017) [45]	GEINO
Glioma anaplastic [24, 56]	SEOM clinical guidelines for anaplastic gliomas (2017)** [54, 83]	GEINO
Glioma low grade [54, 83]	SEOM clinical guideline of diagnosis and management of low-grade glioma (2017)**	GEINO
Head and neck	SEOM clinical guidelines for the treatment of head and neck cancer (2020)*** [46, 84, 85]	TTCC
Hepatic	Diagnostic and treatment for hepatocellular carcinoma. Actualization of the consensus document by AEEH, AEC, SEOM, SERAM, SERVEI and SETH (2021)* [31, 62, 63]	AEEH, AEC, GEMCAD, SERAM, SERVEI, SETH, TTD
Kidney [40, 86–88]	SEOM clinical guideline for treatment of kidney cancer (2019)*** [50, 86–88]	SOGUG
Lung	SEOM clinical guidelines for the treatment of non-small cell lung cancer (2018)* [42, 89]	GECP
	SEOM clinical guidelines for the treatment of small cell lung cancer (SCLC) (2019) [30]	GECP
Lymphoma	SEOM clinical guidelines for the treatment of follicular non-Hodgkin’s lymphoma (2015) [49]	GOTEL
	SEOM clinical guidelines for the treatment of Hodgkin’s lymphoma (2015) [52]	GOTEL
Medulloblastoma	SEOM clinical guideline for management of adult medulloblastoma (2020) [41]	GEINO
Melanoma	SEOM clinical guideline for the management of cutaneous melanoma (2020)* [43, 90, 91]	GEM
Mesothelioma	SEOM clinical guidelines for the treatment of malignant pleural mesothelioma (2020) [47]	GECP
Nasopharynx	SEOM TTCC clinical guideline in nasopharynx cancer (2021)* [53, 92]	TTCC
Neuroendocrine	SEOM clinical guidelines for the diagnosis and treatment of gastroenteropancreatic and bronchial neuroendocrine neoplasms (NENs) (2018)* [37, 93]	GETNE
Ovarian	SEOM clinical guideline in ovarian cancer (2020)* [50, 94, 95]	GEICO
Pancreatic and biliary	SEOM clinical guidelines for pancreatic and biliary tract cancer (2020)* [34, 96, 97]	GEMCAD, TTD
Prostate	SEOM clinical guidelines for the treatment of advanced prostate cancer (2020)*** [36, 98–100]	SOGUG
Sarcoma	SEOM Clinical Guideline of management of soft tissue sarcoma (2020)* [28, 61]	GEIS
Testicular	SEOM clinical guidelines for the management of germ cell testicular cancer (2016) [22]	GG, SGCCG
Thymic	SEOM GECP GETTHI Clinical Guidelines for the treatment of patients with thymic epithelial tumors (2021) [51]	GECP, GETTHI
Thyroid	SEOM clinical guideline thyroid cancer (2019)* [32, 64]	GETNE
Urothelial bladder	SEOM clinical guideline for treatment of muscle‑invasive and metastatic urothelial bladder cancer (2018)*** [35, 101, 102]	SOGUG

*AEC* Spanish Surgeons Association, *AEEH* Spanish Association for the Study of the Liver, *GECP* Spanish Lung Cancer Group, *GEICAM* Spanish Foundation Research Group in Breast Cancer, *GEICO* Spanish Group for Ovary Cancer Investigation, *GEINO* Spanish Group for Neuro Oncology Investigation, *GEIS* Spanish Group for Research on Sarcoma, *GEM* Spanish Multidisciplinary Melanoma Group, *GEMCAD* Spanish Multidisciplinary Group for Digestive Cancer, *GETNE* Spanish Group for Neuroendocrine and Endocrine Tumors, *GETTHI* Spanish Group for Orphan and Infrequent Tumors, *GG* Germinal Oncologic Group, *GOTEL* Oncologic Group for treatment and study of lymphomas, *SERAM* Spanish Society of Medical Radiology, *SERVEI* Spanish Society of Vascular and Interventionist Radiology, *SETH* Spanish Society for Thrombosis and Hemostasia, *SGCCG* Spanish Germ Cell Cancer Group, *SOGUG*: Spanish Oncology Genito Urinary Group, *SOLTI* Spanish Group for the study, treatment and other experimental strategies in solid tumors, *TTCC* Spanish Group for the Treatment of Head and Neck Tumors, *TTD* Digestive Tumor Treatment Group

*thread

**GPC + correction

***thread + correction

Table 2 summarizes the standardized domain scores of the AGREE II and the overall quality rating of the CPGs. As per the pre-defined criteria of this study, 84.8% of CPGs (28) were considered “high quality” [23–28, 30–33, 39, 45, 55–66], 12.0% of CPGs (4) were assessed as “moderate quality” [22, 38, 44, 52, 67–70] and 3.0% CPGs (1) were considered as “low quality” [29]. A moderate agreement was present across all appraisers in this study (average measures ICC = 0.6; 95% CI 0.4, 0.7). Among the six domains of AGREE II in all guidelines, four median scores were rated ≥ 60 (domains 1, 3, 4, and 6). The highest median standardized scores (96.3) were observed in domain 4 (clarity of presentation), whereas domain 5 (applicability) was distinctively low (31.4), with only one of the CPGs scoring above 60%. Regarding domain 3 (rigor of development), the standardized scores ranged from 55.6 to 86.1 (median = 74.3), and 28 of the CPGs scored above 60.0%. The CPG for gastrointestinal sarcomas (GIST) [48] obtained the highest scores, fulfilling 100% of the criteria in two domains, and > 85.0% in domain 3.

**Table 2:** AGREE II standardized domain scores: CPGs by SEOM (n= 33)

Guidelines [references]	Domain (%)						Overall score	Recommendation	Quality^a^
	D1	D2	D3	D4	D5	D6			
Breast (early stage) [23, 55]	87.0	33.3	64.6	88.9	19.4	75.0	4.3	( +)	high
Breast (advanced) [27, 60]	81.5	40.7	61.1	85.2	26.4	75.0	4.3	(+ +)	high
Breast and ovarian [38, 70]	90.7	53.7	59.7	90.7	16.7	77.8	5.0	( +)	moderate
Cervical [29, 82]	46.3	35.2	58.3	87.0	37.5	75.0	4.7	(+)	low
Colorectal (metastasic) [33, 66]	98.1	44.4	79.2	100	23.6	75.0	4.7	(+ +)	high
Colorectal (hereditary) [39, 65]	81.5	42.6	79.9	100	54.2	75.0	5.0	(+ +)	high
Endometrial [25, 57, 58]	57.4	46.3	77.8	88.9	34.7	75.0	5.7	(+ +)	high
Gastric and gastroesophageal junction [44, 67–69]	77.8	48.1	55.6	81.5	15.3	75.0	4.3	( +)	moderate
Gastrointestinal sarcomas [48]	100.0	53.7	86.1	100	48.6	75.0	4.7	(+ +)	high
Glioblastoma [45]	92.6	63.0	77.1	100	77.8	75.0	4.7	(+ +)	high
Glioma (anaplasic) [24, 56]	83.3	40.7	63.9	92.6	11.1	75.0	6.0	(+ +)	high
Glioma (low grade) [54, 83]	92.6	59.3	72.2	100	27.8	75.0	5.0	(+ +)	high
Head and neck [46, 84, 85]	75.9	53.7	70.1	100	13.9	75.0	5.3	( +)	high
Hepatocellular [31, 62, 63]	90.7	63.0	76.4	98.1	31.9	75.0	5.0	(+ +)	high
Kidney [40, 86–88]	87.0	53.7	68.8	100	45.8	75.0	6.0	(+ +)	high
Lung (small cell) [30]	55.6	50.0	70.8	94.4	37.5	80.6	6.0	( +)	high
Lung (non-small cell) [42, 89]	72.2	44.4	68.1	96.3	19.4	75.0	4.3	( +)	high
Lymphoma (follicular non-Hodgkin) [49]	72.2	50.0	77.1	100	15.3	75.0	5.7	(+ +)	high
Lymphoma (Hodgkin) [52]	72.2	50.0	56.3	100	18.1	75.0	5.7	( +)	moderate
Medulloblastoma [41]	90.7	50.0	75.0	98.1	40.3	75.0	6.0	(+ +)	high
Melanoma [43, 90, 91]	75.9	50.0	68.8	98.1	16.7	75.0	4.3	( +)	high
Mesothelioma [47]	81.5	50.0	82.6	100	23.6	77.8	5.0	(+ +)	high
Nasopharynx [53, 92]	87.0	50.0	77.1	100	19.4	75.0	4.7	(+ +)	high
Neuroendocrine [37, 93]	88.9	53.7	74.3	100	33.3	75.0	5.7	(+ +)	high
Ovarian [50, 94, 95]	96.3	53.7	81.9	94.4	33.3	77.8	5.0	(+ +)	high
Pancreatic and biliary [34, 96, 97]	79.6	50.0	63.9	98.1	36.1	75.0	5.7	( +)	high
Prostate [36, 98–100]	90.7	55.6	74.3	96.3	54.2	75.0	5.7	(+ +)	high
Rectal [26, 59]	87.0	46.3	83.3	100	25.0	75.0	5.3	(+ +)	high
Sarcoma [28, 61]	94.4	48.1	84.0	100	59.7	69.4	6.0	(+ +)	high
Testicular [22]	66.7	24.1	59.7	88.9	13.9	69.4	5.7	(+)	moderate
Thymic epithelial [51]	92.6	51.9	80.6	100	34.7	75.0	4.3	(+ +)	high
Thyroid [32, 64]	83.3	40.7	77.1	100	45.8	72.2	5.7	(+ +)	high
Urothelial bladder [35, 101, 102]	96.3	48.1	71.5	100	25.0	75.0	5.7	(+)	high

*D1* Scope and purpose, *D2* Stakeholder involvement, *D3* Rigor of development, *D4* Clarity of presentation, *D5* Applicability, *D6* Editorial independence

^a^CPGs were considered as “high" quality if they scored ≥ 60% in at least three of six AGREE II domains, including domain 3. If three domains or more were assessed with a score of ≥ 60%, except domain 3, they were considered to be of “moderate” overall quality. CPGs scoring < 60% in two or more domains and < 50% in domain 3 were considered as “low" quality

**Fig. 2:**
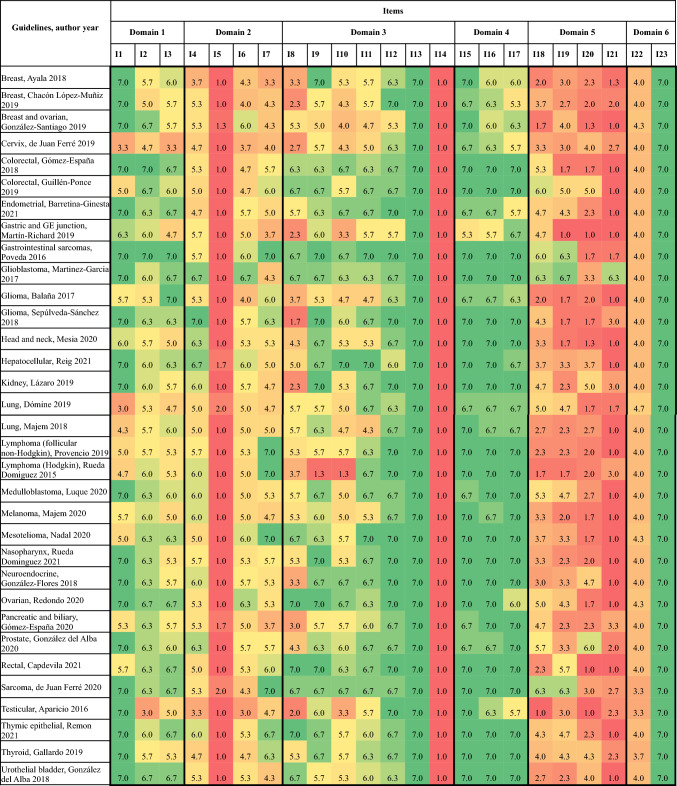
AGREE II item scores of included clinical practice guidelines by the Spanish Society of Medical Oncology (SEOM) (*n* = 33). I1—The overall objective(s) of the guideline is (are) specifically described; I2—The health question(s) covered by the guideline is (are) specifi- cally described; I3—The population (patients, public, etc.) to whom the guideline is meant to apply is specifically described; I4—The guide- line development group includes individuals from all the relevant professional groups; I5—The views and preferences of the target population (patients, public, etc.) have been sought; I6—The target users of the guideline are clearly defined; I7—Systematic methods were used to search for evidence; I8—The criteria for selecting the evidence are clearly described; I9—The strengths and limitations of the body of evidence are clearly described; I10—The methods for formulating the recommendations are clearly described; I11—The health benefits, side effects, and risks have been considered in formulating the recommendations; I12—There is an explicit link between the recommendations and the supporting evidence; I13—The guideline has been externally reviewed by experts prior to its publication; I14—A procedure for updating the guideline is provided; I15—The recommendations are specific and unambiguous; I16—The different options for management of the condition or health issue are clearly presented; I17—Key recommendations are easily identifiable; I18—The guideline provides advice and/or tools on how the recommendations can be put into practice; I19—The guideline describes facilitators and barriers to its application; I20—The potential resource impli- cations of applying the recommendations have been considered; I21—The guideline presents monitoring and/ or auditing criteria; I22—The views of the funding body have not influenced the content of the guideline; I23—Competing interests of guideline development group members have been recorded and addressed

Figure 2 summarizes the item scores of AGREE II. SEOM´s guidelines did not include in their development the views and preferences of the target population as can be inferred from item 5 (1–2, lowest scores). Moreover, they did not clarify the updating methods either, so all obtained the worst score on the Likert scale in item 14 [45]. Also, items 18–21 regarding applicability scored in most cases lower than 4. Specifically, item 21, which refers to monitoring and audit criteria, has a median score of 1.7, being the guideline about glioblastoma the only one with a high score (6.3) in this item [45].

Table 3 summarizes the standardized domain scores of AGREE-REX. Considering only the 28 high-quality CPGs the mean overall AGREE-REX score was 48.5 (SD 11.0) with variability in performance across the individual 9 items. The overall average score of the recommendations was 4.2 out of 7 (SD 0.6). The domain 1 “clinical applicability” got the highest scores (mean 75.8, SD 14.3) and the domain 2  “values and preferences” got the lowest (mean 26.0, SD 12.2). AGREE-REX items that scored the highest were “2. Applicability to Target Users” (mean 6.2; SD 0.7), and “1. Evidence” (mean 5.8; SD 0.5), while the lowest scores were observed for the item “5. Values and Preferences of Patients/Population” (mean 1.6; SD 1.1), and item “6. Values and Preferences of Policy/Decision-Makers” (mean 1.6; SD 1.4).

**Table 3:** AGREE-REX standardized domain scores: CPGs by SEOM (*n*= 28)

Guidelines [references]	Domain (%)			Mean score (SD)
	D1 Clinical applicability	D2 Values and preferences	D3 Implementability	
Breast (early stage) [23, 55]	77.8	45.8	65.5	63.0 (0.2)
Breast (advanced) [27, 60]	72.2	30.6	51.7	41.3 (0.1)
Colorectal (metastasic) [33, 66]	85.2	54.2	20.7	53.4 (0.0)
Colorectal (hereditary) [39, 65]	96.3	25.0	38.0	53.1 (0.4)
Endometrial [25, 57, 58]	35.2	19.4	27.6	27.4 (0.1)
Gastrointestinal sarcomas [48]	92.6	12.5	0.0	35.0 (0.5)
Glioblastoma [45]	98.2	27.8	13.8	46.6 (0.5)
Glioma (anaplasic) [24, 56]	64.8	5.6	34.5	35.0 (0.3)
Glioma (low grade) [54, 83]	87.0	34.7	58.6	60.1 (0.3)
Head and neck [46, 84, 85]	59.3	26.4	58.6	48.1 (0.2)
Hepatocellular [31, 62, 63]	92.6	56.9	82.8	77.4 (0.2)
Kidney [40, 86–88]	77.8	26.4	44.8	49.7 (0.3)
Lung (small cell) [30]	88.9	29.1	31.0	49.7 (0.3)
Lung (non-small cell) [42, 89]	64.8	9.7	0.0	24.8 (0.3)
Lymphoma (follicular non-Hodgkin) [49]	70.4	45.8	62.1	59.4 (0.1)
Medulloblastoma [41]	79.6	27.8	62.1	56.5 (0.3)
Melanoma [43, 90, 91]	85.2	40.3	55.1	60.2 (0.2)
Mesothelioma [47]	92.6	27.8	0.0	40.1 (0.5)
Nasopharynx [53, 92]	75.9	29.2	62.1	55.7 (0.2)
Neuroendocrine [37, 93]	64.8	19.4	41.4	41.9 (0.2)
Ovarian [50, 94, 95]	94.5	37.5	17.2	49.7 (0.4)
Pancreatic and biliary [34, 96, 97]	66.7	27.8	44.8	46.4 (0.2)
Prostate [36, 98–100]	72.2	34.7	44.8	50.6 (0.2)
Rectal [26, 59]	74.1	12.5	37.9	41.5 (0.3)
Sarcoma [28, 61]	75.9	29.2	17.3	40.1 (0.3)
Thymic epithelial [51]	75.9	27.8	69.0	57.6 (0.3)
Thyroid [32, 64]	55.6	27.8	69.0	50.8 (0.2)
Urothelial bladder [35, 101, 102]	87.0	18.1	27.6	44.2 (0.4)

Domain 1 “Clinical applicability”: Items 1.  Evidence, 2. Applicability to Target Users, 3. Applicability to Patients/Population; Domain 2 “Values and Preferences”: Items 4. Values and Preferences of Target Users, 5. Values and Preferences of Patients/Population, 6. Values and Preferences of Policy/Decision-makers, 7. Values and Preferences of Guideline Developers; Domain 3 “Implementability”: Items 8. Purpose, 9. Local application and Adoption.

## Discussion

Overall, this critical review provides a thorough analysis of the quality of CPGs published by the Spanish Society of Medical Oncology on cancer treatment along the last nine years. The study also identified the characteristics of the guidelines, including the types of cancer covered and the timeline of their publication. Ultimately, 33 guidelines were included and assessed, with 28 (85.0%) of those being considered “high quality” according to pre-defined criteria; however, their applicability was found to be poor. One of the main strengths of the guidelines is the domain “clarity of presentation”, in which it achieved the highest possible scores, whereas domain “applicability” was distinctively low (31.4), with only one of the CPGs scoring above 60.0%. SEOM’s guidelines did not include in their formulation the views and preferences of the target population. Moreover, they did not specify the updating methods either.

Our results are pointing out that the SEOM is producing CPGs that meet established standards for methodological rigor. Being high scored in “clarity of presentation” is encouraging because it suggests that the SEOM is effectively communicating its recommendations to clinicians and patients. This result is particularly noteworthy given that clear and understandable guidelines are essential for their effective implementation in clinical practice, ensuring that the recommendations are specific and unambiguous, the different options for management of the condition or health issues are clearly presented, and key recommendations are easily identifiable [73].

However, it is concerning that guidelines scored low in “applicability” because it suggests that may not be as useful for clinicians and patients as they could be. Previous research indicates that for clinical guidelines to have an actual impact on processes and ultimately outcomes of care, they need to be not only well developed and based on scientific evidence but also disseminated and implemented in ways that ensure they are actually used by clinicians [71, 72]. Implementation science frameworks have been used to address challenges in implementing clinical practice guidelines [72]. Also, SEOM's guidelines did not include the views and preferences of the target population or specify the updating methods indicates that there is room for improvement in the guidelines development process. Incorporating patient perspectives into guidelines development can improve the relevance and applicability of guidelines to clinical practice [73]. Specifying updating methods is also important to ensure that guidelines remain up-to-date and reflect the latest evidence [5].

Our critical review used both AGREE II and AGREE-REX, tools used to evaluate practice guidelines, but with different focuses. AGREE II is designed to assess the methodological quality and transparency of practice guidelines, while AGREE-REX is designed to evaluate the clinical credibility and implementability of practice guidelines [17, 20]. AGREE-REX is a complement to AGREE II, rather than an alternative, and provides a blueprint for practice guideline development and reporting. Although scoring high in AGREE II is essential, it does not guarantee that recommendations are optimal for targeted users nor the optimal implementation of the recommendations [19]. In our review, we found that recommendations from guidelines scored as “high quality” on AGREE II, did not align with the values and preferences of their target users, whether they be patients or policy makers, according to AGREE-REX. This result has been previously reported elsewhere [20]. In this context, we consider that guidelines that are developed without considering the values and preferences may not be relevant or applicable to their needs, which can lead to poor adherence and outcomes.

Finally, there have been several critical appraisals of the quality of guidelines in cancer [20, 74–76], but there are no search results that indicate whether ASCO or ESMO have conducted critical appraisals of the quality of guidelines in cancer. One study used the AGREE tool to assess the quality of oncology guidelines developed in different countries [77]. Another study used the AGREE II tool to assess the methodological quality of clinical practice guidelines with physical activity recommendations for people diagnosed with cancer [76]. The results of these studies showed a heterogeneous quality of existing guidelines in cancer, indicating a need for improvement in the development and reporting of guidelines.

### Strengths and limitations

Our research has multiple strengths. We implemented a thorough search strategy to locate SEOM guidelines, and utilized a standardized and globally recognized guidelines appraisal tool (AGREE II). While our study is not the first to critically appraise guidelines [11, 20], it is noteworthy that we are one of the few studies to use the AGREE-REX tool (developed in 2019) for assessing cancer guidelines. Furthermore, to the best of our knowledge, this is one of the first independent evaluations of the quality of cancer treatment guidelines from a scientific society, which adds to the significance of our findings.

Nevertheless, there are also some limitations to our study that need to be acknowledged. First, our study only assessed the methodological quality of the SEOM guidelines and did not evaluate their impact on clinical practice or patient outcomes. Second, while AGREE II and AGREE-REX are recognized appraisal tools, they do have limitations, and therefore, their application alone may not fully encompass all aspects of guidelines quality. Third, we should not be assumed that having a rigorous methodology means that all issues have been dealt with exhaustively and accurately. Some recommendations could not be sufficiently detailed to guide treatment decisions in specific situations, such as advanced cancer, end of life, elderly, and comorbidities. Fourth, we cannot assume that clinicians’ adherence to these guidelines is high, so having high-quality guidelines does not necessarily mean that clinicians are making the right decisions, as many studies previously reported [78–80]. Finally, due to the nature of the study design, our results are limited to the time period of guidelines publication and do not account for any subsequent updates or changes to the guidelines.

It is worth considering the potential redundancy with other cancer treatment guidelines developed by international organizations or societies. While our study focuses on the SEOM guidelines, it is important to acknowledge that other guidelines exist that may provide valuable insights and recommendations. It is worth reflecting on the extent to which these guidelines, taken individually, contribute different nuances and perspectives on cancer treatment and management. In light of this, it is worth asking whether a policy of adapting guidelines [81] might be a more efficient approach than the development of new guidelines from scratch. Such an approach could help to reconcile the differences between guidelines and promote the uptake of best practices across multiple contexts.

### Implications for practice and research

Overall, this review emphasizes the importance of producing high-quality and applicable CPGs in oncology to guide clinical practice and improve patient outcomes. The findings provide insights into the strengths and limitations of SEOM's guidelines and highlight areas where improvement can be made to enhance their relevance and usefulness.

## Conclusions

SEOM guidelines on cancer treatment have been developed with acceptable methodological rigor although they have some drawbacks that could be improved in the future, such as clinical applicability and items regarding patient views and preferences as well as unmeeting policies.

### Supplementary Information

Below is the link to the electronic supplementary material.

Supplementary file1 (DOCX 22 KB)

Supplementary file 2 (PDF 32 KB)

Supplementary file 3 (PDF 43 KB)

## Data Availability

The data used in this study is available upon request. Interested researchers can contact the corresponding author for access to the data.
