# Supplementary material for: Assessing the methodological strengths and limitations of the Spanish Society of Medical Oncology (SEOM) guidelines: a critical appraisal using AGREE II and AGREE-REX tool
Source: Clin Transl Oncol. 2023 Jun 27;26(1):85–97. doi: 10.1007/s12094-023-03219-0 (PMC10761528; doi:10.1007/s12094-023-03219-0)
Supplement: Supplementary file 2 — (PDF 32 KB) [file 12094_2023_3219_MOESM2_ESM.pdf]

Appendix 2: Search strategy for MEDLINE (via PubMed)

(("Neoplasms"[Mesh] OR cancer*[ti] OR carcinom*[ti] OR neoplasm*[ti] OR tumor*[ti] OR tumour*[ti] OR malignan*[ti] OR adenocar*[ti] OR oncolog*[ti]) AND ("Practice guidelines as topic"[Mesh] OR "Practice Guideline"[ptyp] OR practice guideline*[tiab] OR guideline*[ti] OR recommendation*[ti])) AND ("SEOM" [ti] OR "Spain"[ti] OR "Spanish"[ti] OR "Spa*"[ti]) AND ("2014/01/01"[PDAT] : "3000/12/31"[PDAT]))
