# Supplementary material for: Assessing the methodological strengths and limitations of the Spanish Society of Medical Oncology (SEOM) guidelines: a critical appraisal using AGREE II and AGREE-REX tool
Source: Clin Transl Oncol. 2023 Jun 27;26(1):85–97. doi: 10.1007/s12094-023-03219-0 (PMC10761528; doi:10.1007/s12094-023-03219-0)
Supplement: Supplementary file 3 — (PDF 43 KB) [file 12094_2023_3219_MOESM3_ESM.pdf]

Appendix 3. Excluded studies

ID	REFERENCE	REASON
1	https://pubmed.ncbi.nlm.nih.gov/30627983/	wrong publication type
2	https://pubmed.ncbi.nlm.nih.gov/30617923/	wrong intervention
3	https://pubmed.ncbi.nlm.nih.gov/32030655/	wrong publication type
4	https://pubmed.ncbi.nlm.nih.gov/33768441/	wrong intervention
5	https://pubmed.ncbi.nlm.nih.gov/30627982/	wrong intervention
6	https://pubmed.ncbi.nlm.nih.gov/29363023/	wrong publication type
7	https://pubmed.ncbi.nlm.nih.gov/22721791/	wrong publication type
8	https://pubmed.ncbi.nlm.nih.gov/24005836/	wrong period
9	https://pubmed.ncbi.nlm.nih.gov/30607789/	wrong intervention
10	https://pubmed.ncbi.nlm.nih.gov/34303806/	wrong intervention
11	https://pubmed.ncbi.nlm.nih.gov/24026866/	wrong period
12	https://pubmed.ncbi.nlm.nih.gov/23979909/	wrong period
13	https://pubmed.ncbi.nlm.nih.gov/34291418/	wrong intervention
14	https://pubmed.ncbi.nlm.nih.gov/29149431/	wrong intervention
15	https://pubmed.ncbi.nlm.nih.gov/33959901/	wrong intervention
16	https://pubmed.ncbi.nlm.nih.gov/27909872/	wrong publication type
17	https://pubmed.ncbi.nlm.nih.gov/31144212/	wrong publication type
18	https://pubmed.ncbi.nlm.nih.gov/25304221/	wrong intervention
19	https://pubmed.ncbi.nlm.nih.gov/23925725/	wrong period
20	https://pubmed.ncbi.nlm.nih.gov/22721795/	wrong period
21	https://pubmed.ncbi.nlm.nih.gov/27488905/	wrong publication type
22	https://pubmed.ncbi.nlm.nih.gov/22721796/	wrong period
23	https://pubmed.ncbi.nlm.nih.gov/34560242/	wrong intervention
24	https://pubmed.ncbi.nlm.nih.gov/25771086/	wrong publication type
25	https://pubmed.ncbi.nlm.nih.gov/22721792/	wrong period
26	https://pubmed.ncbi.nlm.nih.gov/31346971/	wrong publication type
27	https://pubmed.ncbi.nlm.nih.gov/25392081/	wrong publication type
28	https://pubmed.ncbi.nlm.nih.gov/21821494/	wrong period

29	https://pubmed.ncbi.nlm.nih.gov/22721799/	wrong period
30	https://pubmed.ncbi.nlm.nih.gov/35230619/	wrong intervention
31	https://pubmed.ncbi.nlm.nih.gov/20974570/	wrong intervention
32	https://pubmed.ncbi.nlm.nih.gov/23907290/	wrong period
33	https://pubmed.ncbi.nlm.nih.gov/20974559/	wrong publication type
34	https://pubmed.ncbi.nlm.nih.gov/34500044/	wrong intervention
35	https://pubmed.ncbi.nlm.nih.gov/19531451/	wrong intervention
36	https://pubmed.ncbi.nlm.nih.gov/22721797/	wrong period
37	https://pubmed.ncbi.nlm.nih.gov/29807678/	wrong intervention
38	https://pubmed.ncbi.nlm.nih.gov/21821491/	wrong period
39	https://pubmed.ncbi.nlm.nih.gov/22721798/	wrong period
40	https://pubmed.ncbi.nlm.nih.gov/21821484/	wrong period
41	https://pubmed.ncbi.nlm.nih.gov/23982852/	wrong period
42	https://pubmed.ncbi.nlm.nih.gov/21821485/	wrong period
43	https://pubmed.ncbi.nlm.nih.gov/23896865/	wrong period
44	https://pubmed.ncbi.nlm.nih.gov/31249964/	wrong publication type
45	https://pubmed.ncbi.nlm.nih.gov/24151043/	wrong period
46	https://pubmed.ncbi.nlm.nih.gov/22721794/	wrong period
47	https://pubmed.ncbi.nlm.nih.gov/20974563/	wrong period
48	https://pubmed.ncbi.nlm.nih.gov/21821492/	wrong period
49	https://pubmed.ncbi.nlm.nih.gov/22721800/	wrong period
50	https://pubmed.ncbi.nlm.nih.gov/20974562/	wrong period
51	https://pubmed.ncbi.nlm.nih.gov/23982853/	wrong period
52	https://pubmed.ncbi.nlm.nih.gov/23918288/	wrong population
53	https://pubmed.ncbi.nlm.nih.gov/32006339/	wrong intervention
54	https://pubmed.ncbi.nlm.nih.gov/21821495/	wrong intervention
55	https://pubmed.ncbi.nlm.nih.gov/20974568/	wrong period
56	https://pubmed.ncbi.nlm.nih.gov/20974567/	wrong period
57	https://pubmed.ncbi.nlm.nih.gov/20974564/	wrong period
58	https://pubmed.ncbi.nlm.nih.gov/21821489/	wrong period

59	https://pubmed.ncbi.nlm.nih.gov/20974566/	wrong period
60	https://pubmed.ncbi.nlm.nih.gov/20080468/	wrong period
61	https://pubmed.ncbi.nlm.nih.gov/19451061/	wrong period
62	https://pubmed.ncbi.nlm.nih.gov/20974561/	wrong period
63	https://pubmed.ncbi.nlm.nih.gov/20974569/	wrong period
64	https://pubmed.ncbi.nlm.nih.gov/31981080/	wrong intervention
65	https://pubmed.ncbi.nlm.nih.gov/23150217/	wrong publication type
66	https://pubmed.ncbi.nlm.nih.gov/20974571/	wrong intervention
67	https://pubmed.ncbi.nlm.nih.gov/29138191/	wrong publication type
68	https://pubmed.ncbi.nlm.nih.gov/21821488/	wrong period
69	https://pubmed.ncbi.nlm.nih.gov/29127593/	wrong intervention
70	https://pubmed.ncbi.nlm.nih.gov/29230692/	wrong intervention
71	https://pubmed.ncbi.nlm.nih.gov/20974565/	wrong period
72	https://pubmed.ncbi.nlm.nih.gov/22855192/	wrong publication type
73	https://pubmed.ncbi.nlm.nih.gov/33316363/	wrong publication type
74	https://pubmed.ncbi.nlm.nih.gov/21821486/	wrong period
75	https://pubmed.ncbi.nlm.nih.gov/32650968/	wrong intervention
76	https://pubmed.ncbi.nlm.nih.gov/26680634/	wrong publication type
77	https://pubmed.ncbi.nlm.nih.gov/31993963/	wrong intervention
78	https://pubmed.ncbi.nlm.nih.gov/21821487/	wrong period
79	https://pubmed.ncbi.nlm.nih.gov/25373533/	wrong intervention
80	https://pubmed.ncbi.nlm.nih.gov/21821483/	wrong publication type
81	https://pubmed.ncbi.nlm.nih.gov/21821496/	wrong period
82	https://pubmed.ncbi.nlm.nih.gov/23749327/	wrong intervention
83	https://pubmed.ncbi.nlm.nih.gov/33191439/	wrong publication type
84	https://pubmed.ncbi.nlm.nih.gov/28975575/	wrong publication type
85	https://pubmed.ncbi.nlm.nih.gov/30429069/	wrong publication type
86	https://pubmed.ncbi.nlm.nih.gov/16336087/	wrong intervention
87	https://pubmed.ncbi.nlm.nih.gov/22721801/	wrong period
88	https://pubmed.ncbi.nlm.nih.gov/27896639/	wrong intervention

89	https://pubmed.ncbi.nlm.nih.gov/21821490/	wrong period
90	https://pubmed.ncbi.nlm.nih.gov/27896641/	wrong intervention
91	https://pubmed.ncbi.nlm.nih.gov/30470991/	wrong intervention
92	https://pubmed.ncbi.nlm.nih.gov/27896642/	wrong intervention
93	https://pubmed.ncbi.nlm.nih.gov/32789773/	wrong publication type
94	https://pubmed.ncbi.nlm.nih.gov/21421461/	wrong publication type
95	https://pubmed.ncbi.nlm.nih.gov/25392080/	wrong intervention
96	https://pubmed.ncbi.nlm.nih.gov/25366189/	wrong intervention
97	https://pubmed.ncbi.nlm.nih.gov/28405349/	wrong publication type
98	https://pubmed.ncbi.nlm.nih.gov/25445174/	wrong publication type
99	https://pubmed.ncbi.nlm.nih.gov/25793920/	wrong population
100	https://pubmed.ncbi.nlm.nih.gov/25358801/	wrong population
101	https://pubmed.ncbi.nlm.nih.gov/24019035/	wrong intervention
102	https://pubmed.ncbi.nlm.nih.gov/21821493/	wrong period
103	https://pubmed.ncbi.nlm.nih.gov/27147252/	wrong intervention
104	https://pubmed.ncbi.nlm.nih.gov/34544555/	wrong publication type
105	https://pubmed.ncbi.nlm.nih.gov/22911548/	wrong period
106	https://pubmed.ncbi.nlm.nih.gov/22551539/	wrong intervention
107	https://pubmed.ncbi.nlm.nih.gov/33206333/	wrong intervention
108	https://pubmed.ncbi.nlm.nih.gov/35258806/	wrong intervention
109	https://pubmed.ncbi.nlm.nih.gov/29519710/	wrong publication type
110	https://pubmed.ncbi.nlm.nih.gov/22855150/	wrong intervention
111	https://pubmed.ncbi.nlm.nih.gov/22301407/	wrong publication type
112	https://pubmed.ncbi.nlm.nih.gov/21865135/	wrong intervention
113	https://pubmed.ncbi.nlm.nih.gov/34311246/	wrong intervention
114	https://pubmed.ncbi.nlm.nih.gov/33455693/	wrong intervention
115	https://pubmed.ncbi.nlm.nih.gov/29633183/	wrong publication type
116	https://pubmed.ncbi.nlm.nih.gov/31974819/	wrong publication type
117	https://pubmed.ncbi.nlm.nih.gov/29139040/	wrong population,wrong intervention
118	https://pubmed.ncbi.nlm.nih.gov/20110291/	wrong intervention

119	https://pubmed.ncbi.nlm.nih.gov/26906039/	wrong publication type
120	https://pubmed.ncbi.nlm.nih.gov/24129426/	wrong intervention
121	https://pubmed.ncbi.nlm.nih.gov/28705520/	wrong publication type
122	https://pubmed.ncbi.nlm.nih.gov/29327240/	wrong publication type
123	https://pubmed.ncbi.nlm.nih.gov/34340159/	wrong publication type
124	https://pubmed.ncbi.nlm.nih.gov/23468275/	wrong publication type
125	https://pubmed.ncbi.nlm.nih.gov/32393574/	wrong publication type
126	https://pubmed.ncbi.nlm.nih.gov/22721793/	wrong intervention
127	https://pubmed.ncbi.nlm.nih.gov/29273958/	wrong intervention
128	https://pubmed.ncbi.nlm.nih.gov/25530188/	wrong intervention
129	https://pubmed.ncbi.nlm.nih.gov/33012494/	wrong publication type
130	https://pubmed.ncbi.nlm.nih.gov/34635959/	wrong publication type
131	https://pubmed.ncbi.nlm.nih.gov/26577106/	wrong intervention
132	https://pubmed.ncbi.nlm.nih.gov/29439875/	wrong publication type
133	https://pubmed.ncbi.nlm.nih.gov/29588512/	wrong publication type
134	https://pubmed.ncbi.nlm.nih.gov/21775273/	wrong publication type
135	https://pubmed.ncbi.nlm.nih.gov/28416207/	wrong publication type
136	https://pubmed.ncbi.nlm.nih.gov/30535340/	wrong publication type
137	https://pubmed.ncbi.nlm.nih.gov/19574202/	wrong publication type
138	https://pubmed.ncbi.nlm.nih.gov/19917536/	wrong publication type
139	https://pubmed.ncbi.nlm.nih.gov/16632423/	wrong period
